# Education Research: Establishing a Postgraduate Year-1 Director Enhances Well-Being for Adult Neurology Residents

**DOI:** 10.1212/NE9.0000000000200148

**Published:** 2024-09-09

**Authors:** Robert J. Marquardt, Lindsay A. Ross, Nicolas R. Thompson, Payal Soni, MaryAnn Mays, Andrew B. Buletko

**Affiliations:** From the Department of Neurology (R.J.M., L.A.R., P.S., M.M., A.B.B.), and Department of Quantitative Health Sciences (N.R.T.), Cleveland Clinic Foundation, OH.

## Abstract

**Background and Objectives:**

Adult neurology clinical trainees in Accreditation Council for Graduate Medical Education (ACGME)–accredited residency programs spend their postgraduate year (PGY)-1 within the internal medicine department, potentially causing a perceived disconnect with their neurology program. Our Adult Neurology Clinical Competency Committee found this disconnect may decrease resident well-being. We hypothesized implementing a novel PGY-1 Director role focusing on unique aspects of this first year would improve resident well-being and connection to the neurology program.

**Methods:**

The PGY-1 Director was established as an associate program director in the adult neurology residency program with goals to improve wellness, advocacy, compliance with ACGME requirements, education, and communication. Anonymous surveys compared preintervention (before the PGY-1 Director role) with postintervention resident opinions on PGY-1 experience, assessing wellness, burnout, and perception of advocacy.

**Results:**

A total of 15 (75%) preintervention residents and 23 (96%) postintervention residents completed the study surveys. 53.7% of preintervention residents agreed or strongly agreed to feeling burned out, while only 17.4% of postintervention residents agreed they felt burned out and none strongly agreed. Significant improvement occurred in feeling supported clinically and emotionally and feeling validated. Most postintervention residents felt the PGY-1 Director was valuable and directly led to positive change. The relationship between the neurology and internal medicine departments was improved.

**Discussion:**

A dedicated PGY-1 Director position can improve trainee wellness outcomes and relationships between preliminary and matched departments. This mutually benefits both programs but requires substantial resources. We propose this as a best practice when feasible for ACGME programs with the following suggestions: (1) provide dedicated full-time equivalent time, (2) meet with preliminary program leadership regularly, (3) meet with PGY-1 trainees during orientation and at least quarterly, (4) serve as an advocate, and (5) facilitate mentorship in areas of interest.

## Introduction

The transition into residency, though pivotal, represents one of the most demanding phases in medical training, often associated with significant stress. Most clinical trainees in an Accreditation Council for Graduate Medical Education (ACGME)–accredited medical residency program complete their postgraduate year (PGY)-1, or intern year, within the same institution they will complete the remaining years of residency training, referred to as a categorical training program. Other training programs include a preliminary year that is completed at a different institution before transitioning into the remainder of their chosen residency, referred to as an advanced residency program. In adult neurology, approximately 59% of all programs are categorical, and for either type of program, this year is spent within the department of medicine for a broad clinical experience in general internal medicine, as required by the ACGME.^1,2^

The burnout rate among neurology residents is reported as high as 73%, higher than those of fourth year medical students and all residents at 49% and 51%, respectively.^3-5^ Excessive work hours and increases in workload and responsibility are potential risk factors of burnout that are exacerbated on starting residency.^6-9^ Being in an early training year has also been suggested as an independent risk factor for key features of burnout like feelings of depersonalization and emotional exhaustion.^10^ Depressive symptoms in 1 prospective cohort study increased from 3.9% before internship to 25.7% during internship.^11^ Therefore, it seems particularly relevant to implement strategies focusing on the first year of training.

Programs have implemented various interventions to improve resident well-being, with few focusing on this important, transitional first year. Examples such as introducing wellness curricula, transition courses, and daily mindfulness meditation exercises have shown mixed results.^12,13^ One study surveyed interns at 6 and 12 months after implementing wellness “check-ins” in which a chief resident met with them to discuss personal, professional, and emotional well-being. Interns overall found this helpful for stress management and feeling connected to program leadership.^14^ This may be particularly relevant to neurology interns who spend the first year in a separate department or institution.

At our institution, we aimed to identify the prevalence of this feeling of disconnection, develop an intervention strategy, and assess the effectiveness of this intervention in resident burnout and well-being. We hypothesized implementing a novel PGY-1 Director role focusing on these unique issues would result in decreased PGY-1 burnout, increased sense of well-being, and increased feelings of connection to the neurology program.

## Methods

The need for intervention was considered during the 2020 Adult Neurology Residency Program Clinical Competency Committee (CCC) review of the following data: (1) formal summative feedback through ACGME surveys and wellness surveys identified by program leaders on related questions, (2) formal summative feedback and direct review of comments through graduate medical education (GME) surveys, (3) informal feedback at semiannual resident reviews with the program director, and (4) review of comments from the 2019 and 2020 annual program reviews.

The selected intervention was the development of a PGY-1 Director role, which was established in 2021 as an associate program director (APD) within the adult neurology residency program. Goals were to improve transition into residency, wellness, advocacy, compliance with ACGME requirements, education, and communication and collaboration between potential clinical and/or research mentors specifically to PGY-1 residents.

The following objectives were created to measure the effectiveness and success of this position:

### Primary Objective

1. Improve resident well-being and reduce burnout experienced by PGY-1 neurology residents.

### Secondary Objectives

1. Improve PGY-1 neurology resident perception of advocacy on their behalf.
2. Improve PGY-1 neurology resident perception of the relationship between neurology and internal medicine leadership.

Protected time was provided by the department at a full-time equivalent (FTE) of 0.15. The subsequent commitment included at least quarterly meetings with the PGY-1 trainees in addition to orientation at the beginning of the academic year, attending monthly meetings as the education coordinator with the internal medicine department, and facilitating mentorship with other staff in areas of interest if expressed by the trainee. Education coordinator meetings involved internal medicine education leadership, including the Program Director, chief internal medicine residents, and APDs responsible for education-related endeavors (i.e., curriculum design) in various subspecialties. Topics discussed include all education-related issues, clinical rotation issues, trainee concerns, and ACGME compliance for all internal medicine subspecialties, including neurology.

A literature search was performed in February of 2023 through PubMed, Google Scholar, Academic Search Complete, and Education Research Complete databases for relevant literature pertaining to a PGY-1 Director-type role. Search terms included program director, associate program director, program supervision, PGY-1, postgraduate year-1, and categorical intern, in various combinations.

Anonymous surveys were created (supplemental data 1 and 2) to formally assess the primary and secondary objectives. A 5-point Likert scale was used to assess responses ranging from 1 (strongly disagree) to 5 (strongly agree), with a score of 3 being neutral. Current PGY-3 and PGY-4 residents were included in the preintervention group and current PGY-1 and PGY-2 residents in the postintervention group. Domains assessed included wellness and burnout, preparedness for next level of training, perceived leadership advocacy, openness to feedback, and perceived inclusiveness of both the neurology and internal medicine programs. Surveys were optional and were provided electronically to be completed over a 2-week period.

For both the preintervention and postintervention groups, responses to survey questions were combined and summarized using mean and standard deviation as well as frequency and percent. Among questions that were common to both groups, comparisons were made using an independent samples *t* test for comparing means and the Fisher exact test for comparing percentages.

All computations were performed in R, version 4.2.1.^15^ All tests were 2-sided and alpha set at 0.05. Given the exploratory nature of this study, we did not formally correct for multiple comparisons.

### Standard Protocol Approvals, Registrations, and Patient Consents

This quality improvement project was exempt from Institutional Review Board approval.

### Data Availability

Anonymized data not published within this article will be made available by request from any qualified investigator.

## Results

### Institutional Needs Assessment Data

Based on the 2020 CCC meeting notes and discussion, the annual program evaluation included the following pertinent targeted areas for improvement to better enhance the experience of PGY-1 residents:

1. A renewed focus on the potential for duty hour violations during the PGY-1 year was necessary, along with the development of new strategies to avoid them.
2. The established mentorship program for PGY-2–4 residents needed to be expanded to include the PGY-1 residents.
3. A better working relationship needed to be established between the adult neurology and internal medicine residency programs.

A consensus needs assessment review of GME/ACGME survey comment data from 2019 to 2020 from PGY-1 residents identified the following topics of focus:

1. Difficulty getting involved in research because most residents have not met any staff members in the field of neurology.
2. Generally feeling lost, and without direction, given the lack of a formal mentorship process.
3. Feeling disconnected with the neurology program.
4. Uncertainty as to who was “in charge” of them.

Based on these data, we conducted a formal literature search which did not reveal any relevant publications that directly addressed a similar PGY-1 Director role.

The individual chosen to serve as the PGY-1 Director had additional training and professional development in the following areas: leadership and team building, formal coaching and mentorship, feedback, recognizing signs of burnout and depression, and adult learning theory. This was accomplished using resources already available at the institution, such as the Essentials Program for Health Professions Educators, Distinguished Educator Program, Certificate Program for GME Leadership Teams, and various leadership programs through the American Academy of Neurology.

During the first 2 years, all monthly subspecialty education meetings were attended except one which was missed because of scheduling issues. Two of these meetings involved issues directly pertaining to neurology PGY-1 interns, with one leading to a policy shift ensuring neurology interns had at least as many golden weekends (no clinical duties on both Saturday and Sunday) as internal medicine interns. Other issues that were discussed included duty hour compliance, rotational objectives, and the learning experience/value of each rotation. Each neurology intern also met with the PGY-1 Director a total of 4 times throughout their first year, with 8 desiring additional mentorship in areas of interest which was able to be arranged in all cases.

A total of 15 (75%) preintervention residents and 23 (96%) postintervention residents completed the study surveys. Six residents in the postintervention group responded “Not Applicable” to item 1 (I felt I was well prepared to be a neurology PGY-2 resident). One preintervention resident responded “Not Applicable” to item 6 (I felt I had an advocate in the neurology residency program that I could go to). All other survey items were complete in both groups.

### Primary Outcome

**Figure 1:**
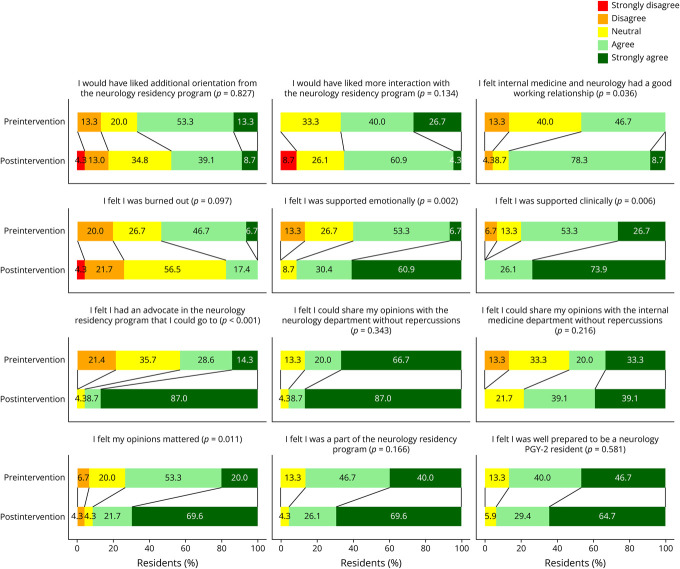
Pre- and Postintervention Trainee Survey Responses to Questions Posed to Both Groups PGY-2 = postgraduate year-2.

**Figure 2:**
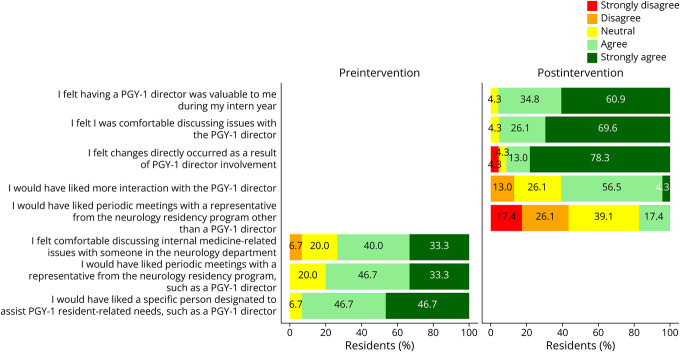
Pre- and Postintervention Trainee Survey Responses to Questions Unique to Each Group PGY-1 = postgraduate year-1.

Among preintervention residents, 53.7% agreed or strongly agreed to feeling burned out, compared with 17.4% of postintervention residents, of which none strongly agreed (Figure 1). This difference did not reach statistical significance (*p* = 0.072). Postintervention residents were more likely to report feeling supported emotionally (*p* < 0.001) and clinically (*p* = 0.006), feeling they had an advocate (*p* < 0.001), and that their opinions mattered (*p* = 0.015). Mean scores for all survey items are given in Table 1. 91.3% felt change occurred directly because of PGY-1 Director involvement (Figure 2).

**Table 1:** Mean (SD) Scores for Survey Items

	Preintervention	Posintervention	Difference in means (95% CI)	*p* Value
I felt I was well prepared to be a neurology PGY-2 resident	4.33 (0.72)	4.59 (0.62)	0.25 (−0.24 to 0.75)	0.297
I felt I was a part of the neurology residency program	4.27 (0.70)	4.65 (0.57)	0.39 (−0.06 to 0.83)	0.088
I felt my opinions mattered	3.87 (0.83)	4.57 (0.79)	0.70 (0.14 to 1.25)	0.015
I felt I could share my opinions with the internal medicine department without repercussion	3.73 (1.10)	4.17 (0.78)	0.44 (−0.24 to 1.12)	0.191
I felt I could share my opinions with the neurology department without repercussion	4.53 (0.74)	4.83 (0.49)	0.29 (−0.16 to 0.74)	0.192
I felt I had an advocate in the neurology residency program that I could go to	3.36 (1.01)	4.83 (0.49)	1.47 (0.86 to 2.08)	<0.001
I felt I was supported clinically	4.00 (0.85)	4.74 (0.45)	0.74 (0.24 to 1.24)	0.006
I felt I was supported emotionally	3.53 (0.83)	4.52 (0.67)	0.99 (0.46 to 1.52)	<0.001
I felt I was burned out	3.40 (0.91)	2.87 (0.76)	−0.53 (−1.11 to 0.05)	0.072
I felt internal medicine and neurology had a good working relationship	3.33 (0.72)	3.91 (0.60)	0.58 (0.12 to 1.04)	0.016
I would have liked more interaction with the neurology residency program	3.93 (0.80)	3.52 (0.95)	−0.41 (−0.99 to 0.17)	0.159
I would have liked additional orientation from the neurology residency program	3.67 (0.90)	3.35 (0.98)	−0.32 (−0.95 to 0.31)	0.311
I felt having a PGY-1 director was valuable to me during my intern year		4.57 (0.59)		
I felt changes directly occurred as a result of PGY-1 director involvement		4.61 (0.94)		
I would have liked more interaction with the PGY-1 director		3.52 (0.79)		
I would have liked periodic meetings with a representative from the neurology residency program other than a PGY-1 director		2.57 (0.99)		
I felt comfortable discussing internal medicine-related issues with someone in the neurology department	4.00 (0.93)			
I would have liked periodic meetings with a representative from the neurology residency program, such as a PGY-1 director	4.13 (0.74)			
I would have liked a specific person designated to assist with PGY-1 resident-related needs, such as a PGY-1 director	4.40 (0.63)			

Abbreviation: PGY = postgraduate year.

### Secondary Outcomes

Figure 1 also shows postintervention residents were more likely to report feeling internal medicine and neurology had a good working relationship (*p* = 0.016) and that they had an advocate (*p* < 0.001). 93.4% of preintervention residents agreed or strongly agreed they would have liked a specific person designated to assist with PGY-1 resident-related needs. Table 2 reflects comments from postintervention surveys about meaningful changes because of the PGY-1 Director role. Most demonstrate appreciation of advocacy and support, consistent with the statistically significant survey findings of postintervention residents perceiving more of these things.

**Table 2:** Postintervention Survey Comments/Responses to “Please Provide 1–2 Comments on Areas a PGY-1 Director Role Was Helpful”

He advocated for us and negotiated with IM leadership
The periodic meetings were helpful and I felt supported
I really enjoyed the 1 on 1 meetings where we could talk about our goals for the year
He was a great advocate for us, making sure we had the same time off as other residents, which previous years didn't have
Having a specific person designated as the point person to go to is incredibly important
I think the IM services and neurology work well together
It was just healthy to have a venting session to someone whose role it is to advocate for change
Most valuable aspect is knowing someone in administration is focused on engaging with PGY1s and our concerns
As an intern it is easy to feel dissociated from the rest of the neurology program, it felt supportive to have this role in place

Abbreviations: IM = internal medicine; PGY = postgraduate year.

Specific comments from preintervention surveys about resident unmet needs during their PGY-1 year also demonstrate a desire for an advocate on their behalf (supplemental data 3).

## Discussion

In this study, we aimed to assess the effect of a novel PGY-1 Director role on PGY-1 resident burnout, well-being, and perceived advocacy. Although the results were not statistically significant, fewer postintervention residents reported feeling burned out compared with preintervention residents. The lack of statistical significance is likely due to our small sample size. Future studies with larger sample sizes are necessary to determine whether such a program can effectively reduce resident burnout. Of interest, although the direct question about resident burnout did not yield statistically significant results, several indirect measures showed meaningful improvement. These included a greater sense of agency with their opinions mattering and emotional support. This suggests that simply asking trainees if they feel burned out is insufficient for screening. It implies that burnout may be incompletely understood by both those who experience it and those who attempt to measure it, especially in relation to mood disorders such as anxiety and depression.^16^

More postintervention residents felt the PGY-1 Director was their advocate and that changes occurred as a result. This is relevant for any program with a preliminary year spent outside the matched specialty. Such a dedicated position might also benefit programs without preliminary years, although this was not directly addressed in this study.

Postintervention residents also had an improved perception of the working relationship between internal medicine and neurology. A critical component of success for this was fostering a strong working relationship with internal medicine program leadership. At our institution, the internal medicine department had a clear desire to work together toward providing a better learning environment for all trainees which was invaluable.

Another key to the success of developing a PGY-1 Director role was acquiring FTE-approved time. This was critical for 2 purposes: first, it demonstrates that resident education and well-being are emphasized at a departmental level; second, it provides adequate time to flexibly meet with the residents and internal medicine department regularly throughout the academic year. These regular face-to-face interactions correlated with residents having a sense of true advocacy. Being able to share their concerns and knowing that change resulted may have improved their sense of control of the learning environment, an important factor in burnout.^17^

Funding new FTE time may be a challenge for many departments. One alternative strategy might include delegating various tasks described herein among leadership already provided FTE time for education-related purposes. Another could be the introduction of a formal coaching program with frequent meetings between interns and core teaching faculty identified through the Clinical Competency Committee. Finally, combining internal medicine and neurology departmental resources to support FTE time or having internal medicine faculty help support mentoring/coaching roles may also take some FTE burden off the neurology program and improve the relationship between programs. Exploring adaptable high-level solutions applicable to various program sizes could enhance the feasibility and effectiveness of implementing similar programs across different contexts.

Finally, our project complements the objectives outlined in the National Plan for Health Workforce Well-being proposed by the National Academy of Medicine, serving as a practical implementation of strategies aimed at enhancing well-being and mitigating burnout of trainees in residency.^18^ It addresses specific priority areas including creating a supportive working environment, investing in research and how to measure well-being, and institutionalizing well-being as a long-term value.

There are limitations to a study of this kind. First, our residency program is large which facilitated the development of a new APD position requiring FTE time. The necessary finances to support such a position also potentially limit the generalizability to programs with more limited resources. This is also true of the background training of the individual who served as the PGY-1 Director. Professional development in key areas was already available which may not be true at other institutions. It should be recognized that although this training is helpful to have, we maintain it should not be required to serve in such a role.

Second, survey data portend response bias, and in our study, the degree of social desirability bias and acquiescence bias cannot be precisely determined. The PGY-1 Director role was generally believed to be a “good idea,” and there was a feeling of wanting it to succeed, which may overestimate its true effect. However, the postintervention improvement seen in contributors to burnout such as perception of the learning environment, feeling engaged with your training program, and mentorship, substantiates the potential of this role. In a similar way, recall bias for the preintervention group may have skewed results, as selective memory of more significant experiences may have influenced their responses more than their true lived experiences in real time.

It is important to point out the deliberate decision of the survey to be optional, the aim of which was to respect inherent power dynamics between program leadership and trainees. Requiring mandatory participation in a survey in which trainees are evaluating an APD may introduce coercion and undermine trust and should therefore be avoided.

Finally, there was a leadership change in the internal medicine program that shifted the educational culture and facilitated many of the PGY-1 Director goals. Although important, this does not fully account for the enhanced sense of advocacy reported by postintervention residents. Wellness activities were also incorporated into educational conferences once per month within the neurology residency and may have affected burnout. Topics covered included nutrition, mental health resources, exercise, and work-place stressors. However, owing to scheduling constraints, PGY-1 residents cannot regularly attend these activities thereby limiting their effect on burnout.

In summary, trainee wellness outcome measures improved after the implementation of a PGY-1 Director role. When feasible, we propose a dedicated PGY-1 director role as a best practice for categorical ACGME training programs with a preliminary year with the following suggestions:

1. The PGY-1 Director role should have dedicated FTE time. We propose 0.10 FTE with more, or less, being necessary depending on the size of the training program. If additional FTE time cannot be allocated, the PGY-1 role should still be emphasized as a critical role within the program using the existing FTE allotment. Programs who find such a change too difficult or impractical should explore alternative solutions that are better suited for their unique situation.
2. Meet with leadership from the program the interns are currently training in at least biannually to review duty hours, curriculum goals, and strategies for improvement.
3. Meet with interns during their orientation to review the position goals.
4. Meet with interns at least quarterly on a mandatory basis to review their progress, inquire about their well-being, and provide updates from leadership of both training programs. Additional meetings or referral for psychological care on an opt-out basis may be additionally warranted.^19^
5. Serve as an advocate with an open-door policy.
6. Facilitate mentorship between interns and professional staff in areas of potential interest. This should be performed at the intern's request if, in the PGY-1 Director's view, it will not produce undue additional burden on the intern. This should be monitored at subsequent meetings.

## Appendix Authors

**Table:**

Name	Location	Contribution
Robert J. Marquardt, DO	Department of Neurology, Cleveland Clinic Foundation, OH	Drafting/revision of the manuscript for content, including medical writing for content; major role in the acquisition of data; study concept or design; analysis or interpretation of data
Lindsay A. Ross, MD	Department of Neurology, Cleveland Clinic Foundation, OH	Drafting/revision of the manuscript for content, including medical writing for content; analysis or interpretation of data
Nicolas R. Thompson, MS	Department of Quantitative Health Sciences, Cleveland Clinic Foundation, OH	Drafting/revision of the manuscript for content, including medical writing for content; analysis or interpretation of data
Payal Soni, MD	Department of Neurology, Cleveland Clinic Foundation, OH	Drafting/revision of the manuscript for content, including medical writing for content
MaryAnn Mays, MD	Department of Neurology, Cleveland Clinic Foundation, OH	Drafting/revision of the manuscript for content, including medical writing for content
Andrew B. Buletko, MD	Department of Neurology, Cleveland Clinic Foundation, OH	Drafting/revision of the manuscript for content, including medical writing for content; study concept or design

## Glossary

ACGME: Accreditation Council for Graduate Medical Education
APD: associate program director
CCC: Clinical Competency Committee
FTE: full-time equivalent
GME: graduate medical education
PGY: postgraduate year
